# Early Life Stress and Child Temperament Style as Predictors of Childhood Anxiety and Depressive Symptoms: Findings from the Longitudinal Study of Australian Children

**DOI:** 10.1155/2011/296026

**Published:** 2011-11-13

**Authors:** Andrew J. Lewis, Craig A. Olsson

**Affiliations:** ^1^School of Psychology, Faculty of Health, Medicine, Nursing and Behavioural Sciences, Deakin University, Melbourne 3217, VIC, Australia; ^2^Murdoch Childrens Research Institute, Royal Children's Hospital and The University of Melbourne (Psychological Sciences and Department of Paediatrics), Parkville 3052, VIC, Australia

## Abstract

*Objective*. The purpose of this study was to determine whether the relationship between stressful infant environments and later childhood anxiety and depressive symptoms varies as a function of individual differences in temperament style. *Methods*. Data was drawn from the Longitudinal Study of Australian Children (LSAC). This study examined 3425 infants assessed at three time points, at 1-year, at 2/3 years and at 4/5 years. Temperament was measured using a 12-item version of Toddler Temperament Scale (TTS) and was scored for reactive, avoidant, and impulsive dimensions. Logistic regression was used to model direct relationships and additive interactions between early life stress, temperament, and emotional symptoms at 4 years of age. Analyses were adjusted for socioeconomic status, parental education, and marital status. *Results*. Stressful family environments experienced in the infant's first year of life (high versus low) and high reactive, avoidant, and impulsive temperament styles directly and independently predicted anxiety and depressive problems in children at 4 years of age. There was no evidence of interaction between temperament and family stress exposure. *Conclusions*. Both infant temperament and stress exposures are independent and notable predictors of later anxiety and depressive problems in childhood. The risk relationship between stress exposure in infancy and childhood emotion problems did not vary as a function of infant temperament. Implications for preventive intervention and future research directions are discussed.

## 1. Introduction

Children as young as three years of age have been shown to meet DSM IV criteria for major depressive disorder [1]. Childhood onset of depressive symptoms has been associated with a distinct pattern of risk factors while childhood depression is itself a major risk factor for the recurrence of depression in adulthood [2, 3]. Among the most well-characterized risk factors are stress exposure (antenatal and perinatal) and patterns of emotion dysregulation in infancy indicated by temperamental dispositions towards avoidance, impulsivity and stress reactivity [4–6]. The determinants of early childhood depressive symptoms are of interest for clinical and preventative interventions. In this study, we will focus on early temperament and stressful life events, separately and in interaction, as predictors of anxiety and depressive problems in early childhood and draw on life course data to examine developmental pathways toward depressive symptoms. 

### 1.1. Childhood Temperament as a Factor in Emotional Regulation

One of the most influential theories of temperament is Cloninger's model grounded in genetic, psychobiologic, and evolutionary theory which informs a broad theory of personal and moral development as well as vulnerability to psychological disorder [7–9]. The role of temperament in Cloninger's model is not dissimilar to that of other theories of temperament insofar as temperament is considered to reflect individual differences in the regulation of experience which emerges early in life and remain moderately stable across development. Temperament is distinct from character which develops in a stepwise manner over the life course, progressively assimilating higher-order cognitive capacities, and experience-dependent social and cultural learning, leading to increasingly sophisticated representations of the self over time. Temperament is a highly heritable platform for such development but remains open to interaction with the environment across development. 

Cloninger's model of temperament is measured using four dimensions: novelty seeking (NS), harm avoidance (HA), reward dependence (RD), and persistence (P) [9]. Novelty seeking refers to a tendency to respond strongly to novel stimuli and to avoid monotony or potential punishment (and has been linked to dopaminergic activity). Harm avoidance is conceptualized as a tendency to show high reactivity to aversive stimuli leading to the inhibition of behavior (and has been associated with serotonergic activity). Reward dependence indicates a tendency to maintain behavior which has previously been associated with reward (and been related to noradrenergic activity). Persistence indicates a tendency towards perseverance of effort despite frustration but has not been linked to a specific monoamine neurochemistry. More recent animal studies have suggested considerable overlap in the monoamine neurochemistries underlying temperament [10, 11] while considerable research is currently emerging with respect to genetic variants mediating neurotransmitter function in circuitry involved in mood disorder and temperament [12].

Much of the research on Cloninger's biopsychological model of temperament and personality has been undertaken with respect to adult psychopathology. The measurement of temperamental dimensions prospectively in early childhood as a predictor of subsequent emotional symptoms within a longitudinal cohort design has been recommended in several papers [13, 14]. The development of the preschool temperament and character inventory (psTCI) enabled Cloninger's dimensions to be examined in children and the results compared to other measures of temperament in childhood [14]. The current study used a shortened version of a related measure, the Toddler Temperament Scale (TTS) which measures approach, reactivity, and persistence dimensions of temperament. Examination of items from the psTCI and the TTS shows very similar wording for items measuring HA, NS, and P, respectively. 

There is substantial evidence of a role for childhood temperament in the aetiology of emotional symptoms in early childhood. In a sample of 30-month-old children using the psTCI, Constantino et al. [14] reported an association between internalizing and both HA (*r* = .49) and NS (*r* = .66) but a negligible association with persistence. Using other measures of temperament, highly reactive infants were found to be more likely to display anxious symptoms in mid childhood (7 years) [5]. Avoidance at 2.5 years has been found to predict anxiety in early adolescence (12-13 years) and emotionality at both 18 months, and 2.5 years of age are associated with anxiety and depression in early adolescence [6]. Using the Dunedin Multidisciplinary Health and Development Study, a particularly long running study, avoidance in childhood was used to predict risk for a diagnosis of depression at age 21 years [15]. The consistency of findings on both HA and NS temperament as predictive of anxiety and depressive symptoms across differing research methodologies (i.e., measurement tools, time spans, and ages) illustrates the significance of early temperament in psychological development.

### 1.2. Stress in Early Childhood as a Factor in Emotional Regulation

Environmental stresses have been widely investigated as a source of anxiety and depressive problems in children and can be divided into stresses occurring as a result of (1) parental relationship dysfunction, (2) parent-child interaction, (3) socioeconomic disadvantage, or (4) negative life events [16]. Here we focus on negative life events occurring in early life as one of the most consistent predictors of anxiety and depressive problems across the life course. Increasingly, the application of ideas derived from the developmental origin of health and disease (DOHaD) hypothesis is suggesting a higher degree of vulnerability to stressors which occur within the antenatal and early postnatal environment [17]. Van den Bergh's review of 14 prospective studies suggested that antenatal maternal stress creates risk for behavioural and emotional regulation problems in children [18]. Theories derived from this body of literature focus on the early development of the HPA axis and the links between HPA dysregulation and vulnerability to anxiety and depressive disorders [19–21].

### 1.3. Interaction between Infant Temperament and Stress Exposure

Since temperament regulates a child's personal experience of the external world, response to stressful life events might be expected to vary as a function of temperament style. In principle, temperament should interact with an individual's appraisal of a given stressor, the degree of stress experienced, efforts to cope with stress, and psychobiological correlations of the response to stress [22]. However, little is known about interaction between infant temperament and stress exposure in creating risk for anxiety and depressive problems in early childhood. Yet temperament can be regarded as a behavioral proxy for heritable differences in stress regulation and is a potentially important driver of sensitivity to social challenges such as family life stressors and maternal depression. Greater knowledge of person-by-environment interactions therefore holds considerable promise for identifying at-risk populations and aligning psychosocial resources for effective and targeted prevention.

### 1.4. Aims and Hypotheses

The purpose of the study was to examine interaction between infant temperament and stress exposure in the development of anxiety and depressive symptoms in childhood (4- to 5-years) using data from 3425 children participating in the Longitudinal Study of Australian Children (LSAC). Specifically, the aims were to examine prediction of anxious-depressive symptoms in childhood as a function of (1) infant stress exposure, (2) infant temperament (avoidant, impulsive, reactive), and (3) interaction between infant temperament and stress exposure. We sought to test the hypothesis that both stressful life events experienced in the first year of life and high reactive, avoidant, and impulsive children (high temperament risk) would directly and independently predict anxious-depressive symptoms in children at 4 years of age. We further hypothesized that high reactive, avoidant, and impulsive temperament styles would interact with early life stressors to augment risk anxious-depressive symptoms.

## 2. Methods

### 2.1. Study Design and Sample

Data was drawn from the first three waves of the Longitudinal Study of Australian Children (LSAC), a nationally representative study of the growth and development of children in Australia. LSAC was initiated by the Australian Government Department of Families, Housing, Community Services, and Indigenous Affairs. The sampling design and method have been described in Soloff et al. [23]. LSAC used a two-stage cluster sampling design with Australian postcodes (stratified by state of residence and urban versus rural status) as the primary sampling units. Secondary sampling units were infants born between March 2003 and February 2004 selected from the Australian Medicare database. Random selection of infants within each postcode produced a cohort aged between 3 and 19 months, with all birth months represented. Of those selected infants who were contacted, 5107 parents elected to take part in the first wave of LSAC in 2004 (64.2% response rate). Wave two data was collected in 2006, and wave three commenced in 2008. The sample for this current analysis was limited to the 3425 children who had complete data for the Strengths and Difficulties Questionnaire delivered in Wave 3 of the study at the 4-5-year time point.

### 2.2. Procedures

Data was collected from the child's primary caregiver via face-to-face interview with a trained researcher. 98.6% of primary caregivers were the child's mother [23]. After each interview, both primary and secondary caregivers completed a self-report questionnaire. The study was approved by the Australian Institute of Family Studies Ethics Committee, and a parent provided written informed consent for every participant.

### 2.3. Measures

#### 2.3.1. Demographic Data

Mothers were asked to report on their child's gender and age in months as well as their own age in years, country of birth (Australia/New Zealand versus other), marital status (married versus other), main language spoken at home (English versus other), and employment status (part time/variable work hours versus full time). Social and economic disadvantage was measured using Socioeconomic Indexes for Areas (SEIFA) which is based on Australian census data (Australian Bureau of Statistics, 2001). The index of relative socioeconomic disadvantage uses postcode of residence to determine neighborhood economic status and has been standardized to a mean of 1000 (SD 100), with higher values indicting a greater advantage.

#### 2.3.2. Temperament

This study makes use of a shortened version of the Australian revision of the Toddler Temperament Scale (TTS). This is a highly regarded and frequently used questionnaire which is a psychometrically sound measure of early childhood behaviour [4]. The TTS is a 97-item measure which was first implemented in the Australian Temperament Project in 1983. Items in the TTS are typically grouped into six temperament styles which have moderate-to-high internal consistency (alphas = 0.53−0.76) and good test-retest reliability [24]. The shortened TTS used in LSAC includes 4 items each for approach, persistence, and reactivity rated on a six-point scale (alphas = 0.98−0.99). For the current analysis, each temperament style was dichotomized into high/low. These were calculated by dividing the distribution into three equal groups with high scores taken as the top third of the distribution for reactive, and the bottom third of the distribution for persistence (to form “impulsive”) and approach (to form “avoidant”). Risk temperament styles were compared to the remaining two thirds of the distribution. For comparison to Cloninger's terminology, the TTS dimension of reactive is analogous to novelty seeking, persistence refers to the same dimension and avoidant is analogous to Cloninger's harm avoidance.

#### 2.3.3. Early Life Stress

At the first wave of the study, when children were between 0-1 years of age, parents indicated exposure to adverse life events over the past year. As such this period of time covers the late antenatal period and postpartum period of the child's life. Participants indicated the exposure to such life events (yes or no) from a list of 13 items which included marital breakdown, serious illness or death of friend or relative, employment or workplace stressors, relationship conflict, and substance use. These were summed and dichotomised. Following Rutter's observations regarding the deleterious impact of cumulative stressful life events, for the current analysis, high-stress environments were considered to be those in which parents indicated that they had experienced two or more of these stressful life events [25].

#### 2.3.4. Anxiety and Depressive Symptoms

Anxiety and depressive symptoms were measured from the emotional symptoms subscale of the Strengths and Difficulties Questionnaire (SDQ) [26]. The SDQ is a 25-item measure of behavioral and emotional problems for children aged 4 to 16 years which is widely used and has sound psychometric properties. The anxiety and depressive symptoms scale has five items which are rated 1 = not true; 2 = somewhat true; 3 = certainly true. The mean of the 5 items is used as a summary score. Items assess anxiety and depressive symptoms of somatic complaints, worried, unhappy or tearful, nervous or lacking confidence, and fearful. In the current study, a dichotomised score (high/low) was created by taking high scores to be those top decile (10%).

#### 2.3.5. Covariates

Information was collected about several factors which may potentially confound the relationship between early life stress and anxiety and depressive symptoms. For this study, we examined differences between children with and without anxiety and depressive symptoms at 4-5 years of age in terms of gender, family structure (married versus other), and indicators of socioeconomic status (SEIFA score as a continuous measure as described above). We also examined ethnicity as a potential covariate codes in terms of Australian/New Zealand origin versus other.

### 2.4. Statistical Analysis

All analyses were performed using the SPSS version 18 (SPSS Inc, Chicago, Ill, USA). Those with missing data on all of the key variables were excluded listwise from the analyses. Population weights were used in all adjusted analyses.

We examined the joint effect of temperament (reactive, persistent, and approach) and early life stress on risk for anxiety and depressive problems in childhood based on the additive scale and using a 2 × 4 table format with a single common reference group [27, 28]. This approach differs from conventional models of interaction based on the multiplicative scale and using at least two reference groups (one for each level of the moderating variable). The 2 × 4 approach provides easily accessible information on the independent and joint effects of each risk factor with respect to a reference group defined by exposure to neither risk factor. We defined four composite exposures: (level 1) high temperamental risk and high social stress (joint effects), (level 2) high temperamental risk and low social stress (temperamental risk only), (level 3) low temperamental risk and high social stress (social risk only), and (level 4) low temperamental risk and low social stress (reference group). 

Joint effects were examined by comparing risk associated with the joint exposure to both temperament and social stress factors (level 1) and risk associated exposure to neither factor (level 4, references group). However, joint exposure does not necessarily mean that both temperament and life stress processes are acting together within one causal mechanism. To estimate the percentage of risk due to the combined actions of both exposures, we first summed risks at level 2 (temperamental risk only) and level 3 (early life stress risks only) and then subtracted the background risk (level 4) to obtain the *expected risk* for no interaction. The difference between the *expected risk for no interaction* and the *observed risk for joint exposure* was then divided by the *observed risk for joint exposure *to represent the % risk attributable to the joint action of both exposures. Interaction is notable when the % risk attributable to joint interaction exceeds 30% [29].

Within each exposure level, we estimated the positive predictive value (PV+) and the attributable risk percent (AR%, also referred to as the attributable fraction in the exposed). PV+ is the probability of reporting problematic anxiety and depressive symptoms given exposure status and provides information of value for *prediction* of individual level risk. AR% is the proportion of individuals showing anxiety and depressive symptoms within a particular exposure level that is attributable to having that exposure (*cf*. reference group).

## 3. Results

### 3.1. Sample Characteristics

Baseline characteristics were examined from the first wave of the study for those children and parents who reported on anxiety and depressive symptoms at the third wave of the study. Sample characteristics were examined including gender, age of parent and child, parental education, ethnicity, and socioeconomic status. Differences between categorical and continuous data for these variables for the groups with and without anxiety and depressive symptoms were examined. Significant differences were discerned using a Chi square or independent samples *t*-test as appropriate and results are shown in Table 1. Significant differences were found for mothers of children in the anxiety and depressive symptoms group being slightly younger and less likely to be married.

### 3.2. Infant Temperament, Infant Stress Exposure and Childhood Anxiety and Depressive Maladjustment

Association between early life stress in the first year of life, child temperament at 2 years of age, and anxiety and depressive symptoms at 4 years of age were tested using logistic regression. In the direct model, each variable was entered simultaneously into the regression model, and results are therefore controlled for the effect of the other predictors. Risk relationships were observed for all predictor variables (Table 2). Notably, the odds of reporting anxious-depressive symptoms in childhood were doubled in those with high reactive and avoidant temperament styles. More marginal elevations in the odds of reporting childhood anxiety and depressive problems (~20 to 40%) were observed for high impulsivity and stress exposure, respectively. In the second step of the logistic regression analysis, interaction between gender and temperament and gender and stress were examined. There was evidence of an interaction between gender and avoidant temperament in prediction of anxious-depressive symptoms; specifically, that the relationship between avoidant temperament styles and anxious-depressive symptomes was higher for boys than girls. There was no evidence of interaction between gender and either reactive or impulsive styles. 

There was no evidence of infant temperament by stress exposure correlations. Tables 3, 4, and 5 show no evidence of interaction between temperament and stress exposure in prediction of anxious-depressive symptoms in childhood for any of the three temperament styles examined; however, interaction between infant reactivity and stress exposure was close to being noteworthy (28%). Odds ratios and PV+ estimates were highly consistent across each risk level: temperament only OR: 1.2–1.9/PV+ 10–12%, social stress only OR: 1.3–1.4/PV+ 9–11%, and interaction OR: 1.9–3.0/PV+ 14–19%. AR% was higher than PV+ in all cases; however, AR% for infant impulsivity was lower than for reactive and avoidant temperaments (19% cf. 47-48%). 

## 4. Discussion

The purpose of the study was to examine independent and interactive effects of early stressful life events and temperament style in the development of anxiety and depressive symptoms in early childhood. Results support the notion that stressful family environments experienced in the infant's first year of life and high reactive, avoidant, and impulsive temperament styles directly and independently contribute to anxiety and depressive symptoms in children at 4 years of age. However, contrary to expectations, we observed no notable interaction between temperamental and social risks. This study does not support the hypothesis that temperament style creates underlying patterns of individual susceptibility to social risks for later emotional disorders. 

The central question in the current paper concerns the possible interaction between environmental factors inducing stress and heritable individual differences in temperament. Both experimental studies in animals and naturalistic studies of children raised in adversity have shown that severe perturbations in the family environment such as maternal deprivation and significant maltreatment can produce derangement of the normal relationships between monoamine neurotransmitters [30, 31]. The current study examines more modest perturbations of the family environment consistent with degrees of early life stress exposure which are relatively common in Western populations.

Temperament is generally understood to refer to emotional or affective aspects of the developing personality [32]. This relationship between emotional regulation and the development of anxiety and depressive symptoms emerged strongly as our two-year-old temperament measures uniquely and independently predicted later mood-related symptoms 4-5-year children. This result confirms the predictive validity and clinical significance of temperament as an early risk factor indicating vulnerability for childhood onset depressive symptoms. 

While a number of studies have found associations between Cloninger's temperament dimension of harm avoidance and depression, these studies have largely been conducted with clinical adult populations [13, 33–35]. The current finding suggests an extension within a child sample of Cloninger's finding in an adult population sample that HA is predictive of depression [36]. Findings with respect to Cloninger's novelty seeking (NS), considered here to be analogous to reactivity, have been mixed. While Celikel et al. [13] did report an association between NS and depression, there have been several studies which have not found such an association [37, 38].

The current findings are interesting to consider in the light of the idea that temperament is one of the relatively stable characteristics to emerge early in the development of personality across the life course [39]. It is also widely acknowledged that temperament interacts with life course factors to moderate continuity and change in personality across development [9, 40]. However, the current study suggests that the very early experience of a moderate level of life stress within the family environment does not substantially interact with the temperament styles measured in the current study. This is consistent with Cloninger's assertion that temperament is relatively immune from the influence of culture or social experience [41].

It is also to be noted that our results point to an interaction between gender and temperament in prediction of anxiety and depressive symptoms, showing that boys with avoidant temperament are particularly susceptible. In a recent meta-analysis, negative affectivity did not show significant gender differences with the small gender difference in fear (*d *= 0.12) not being sufficient to conclude that boys and girls differ markedly in fearfulness [42]. Our finding that boys with avoidant temperament are more vulnerable than girls suggests that there may be a subset of boys whose avoidant temperament predisposes them to negative social experiences and negative self appraisal.

Our interest in interaction is based on a desire to understand modifiable factors in early development which could be a target for preventive intervention. Despite marked improvements in knowledge of individual risk factors for childhood anxiety and depressive problems, the efficacy of most universal (school-based) preventive interventions designed to minimize risk exposure remains unremarkable [43]. Targeted approaches to preventive intervention appear to hold greater promise [43]; however, they remain fundamentally limited by a general lack of knowledge about individual differences in sensitivity to stressful life events (person-by-environment interaction). From this applied perspective, the current results suggest that investment should be targeted at developing independent and tailored preventive intervention aimed at minimising risk associated with both temperamental and social risk factors for anxious-depressive symptoms in early childhood. However, further research is needed to identify social exposures that are capable of buffering constitutional factors. Such exposures may well be factors which have a more direct impact on the child such as parental mental health, family conflict, and hostile parenting styles.

This study presented a unique opportunity to test such models in several respects. Very few large population studies in children have the scope to examine the interactions of both life stressors and temperament across early childhood using several different modeling techniques. Sample size also permits robust testing of differences between male and female children in the cohort in a nationally representative sample with relatively low attrition across three waves of data collection. As a population-based study, this also includes inevitable limitations in terms of the use of brief measures of anxiety and depressive symptoms and parental report versions of temperament and life stress variables. The life stress measure can only act as an indicator of environmental events which are assumed to lead to infant stress exposure but without a physiological indicator of stress reactivity; this remains only an assumption. Our study was also based on an assumption that what we regarded as a moderate degree of stress exposure would be sufficient to find both direct and interactive effects. In addition, the study design asked parents to rate stress over the last year while their infants were within their first year, thereby, not enabling a clear demarcation between antenatal and postnatal stressors nor precision in the time of stress exposure. Studies vary widely in terms of the level, timing, and type of infant stress exposure so this suggests that future studies and reviews can examine different timing, levels, and types of infant stress exposure. Finally, it should be noted that we examined anxious and depressive symptoms only at one-time point which does not rule out the possibility that the interaction between early life stress and child temperament may be discerned at a later point in development.

Our emphasis in this study has been to investigate infant stress exposure and temperament as predictors of early childhood indicators of anxiety and depressive symptoms. Major environmental adversity such as maternal deprivation has been repeatedly shown to be capable of overriding temperament. Our findings indicate that temperament styles are considerably stronger predictors of such anxiety and depressive symptoms than exposure to a moderate level of early life stress. We have found that moderate environmental stressors in the family environment as a whole seem to have little or no interaction with temperament and allow for the persistence of temperament influence in early child adjustment. Such findings suggest that temperament requires a “species typical” family social environment in order to influence the direction of child development, but it is also reasonably robust to moderate environmental perturbation. Our findings also suggest a differential susceptible to avoidant temperament as a risk factor for anxiety and depressive symptoms specifically in boys supporting previous findings suggestive of sex-specific gene × environment interactions with temperament operating across the developmental life course [44].

## Figures and Tables

**Table 1:** Demographic characteristics of participants (*N* = 3824).

Characteristic	Total	No anxiety and depressive symptoms	Anxiety and depressive symptoms	*P* value
	*N* (%)	*N* (%)	*N* (%)	
Total	3824	3444	380	
Child at time 1				
Age (months), M (SD)	8.77 (2.5)	8.75 (2.5)	8.91 (2.6)	0.29
Male	1972 (51.6)	1798 (51.9)	183 (48.2)	0.16
Maternal at time 1				
Age (years), M (SD)	31.5 (5.2)	31.6 (5.2)	30.6 (5.1)	<0.01
Born in Australia/New Zealand	3200 (83.7)	2889 (83.9)	311 (81.8)	0.31
Married	2962 (77.5)	2687 (78.0)	275 (72.4)	0.01
Did not complete yr 12 high school	507 (13.3)	449 (13.0)	58 (15.3)	0.39
Disadvantage index (SEIFA), M (SD)	1011 (59.9)	1011 (59.4)	1007 (64.1)	0.19

**Table 2:** Summary of logistic regression analysis predicting anxiety and depressive symptoms in 4 year old children.

	Anxious-depressive symptoms at 3-4 years of age, *N* = 314 (10.0%)	Model 1 unadjusted	Model 2 adjusted	Model 3 (males)	Model 4 (females)
	*n* (%)	OR (95% CI)	aOR (95% CI)	aOR (95% CI)	aOR (95% CI)
High life stress *N* = 1132 (33.7%)	135 (11.9)	1.37 (1.06, 1.77)	1.31 (1.02, 1.71)	1.18 (0.81, 1.73)	1.47 (1.02, 2.11)
High reactive *N* = 742 (23.7%)	120 (16.2)	2.03 (1.56, 2.46)	1.97 (1.51, 2.57)	1.84 (1.25, 2.71)	2.07 (1.43, 3.00)
High avoidant *N* = 1131 (36.1%)	156 (13.8)	1.85 (1.44, 2.38)	1.85 (1.43, 2.38)	2.50 (1.74, 3.60)	1.34 (0.94, 1.91)
High impulsive *N* = 1226 (39.1%)	132 (10.8)	1.20 (0.93, 1.55)	1.23 (0.96, 1.59)	1.24 (0.86, 1.78)	1.28 (0.89, 1.85)

Note: *N*: Number at risk; *n*: Number with depression level endpoint; %: prevalence.

Model 1: unadjusted odd ratio (OR), 95% confidence interval (CI) for associations between temperament and anxious-depressive symptoms in childhood.

Model 2: aORs adjusted also for maternal age, parent's marital status, and SEIFA index of socioeconomic status.

Models 3 and 4: aORs adjusted for the same covariates separately for males and females.

**Table 3:** Odds ratios for anxiety and depressive symptoms at age 4 years attributable to person-environment interaction between early life stress and reactive temperament style.

		Anxiety and depressive symptoms at 4 years							
Infant reactivity	Stress exposure		Noncase	Case	OR^†^	95% CI^†^		PPV	AR%
Low	Low	Reference	1405	113	1.0				
Low	High	ORe^†^	620	64	1.3	(0.96-1.5)		9%	22%
High	Low	ORp^†^	360	54	1.9	(1.3-2.6)		13%	47%
High	High	ORpe^†^	200	48	3.0	(2.1-4.3)		19%	66%
Total			2585	279					
			Expected ORpe		Departure from		% of ORpe attributable to the joint		
			assuming no joint action (E)		expected (DE)		action of person and environment		
	Additive model of interaction		ORe+ORp-1		ORpe-E		DE/ORpe		
			2.2		0.8		28%		

^†^OR: odds ratio, 95% CI: 95% confidence interval, ORe: lnfant stress exposure, ORp: infant temperament, ORpe: infant temperament and stress exposure, Reference: neither exposure; AR%: attributable risk percent, PPV: positive predictive value.

**Table 4:** Odds ratios for anxiety and depressive symptoms at age 4 years attributable to person-environment interaction between early life stress and avoidant temperament style.

		Anxiety and depressive symptoms at 4 years								
Infant avoidance	Stress exposure		Noncase	Case	OR^†^	95% CI^†^		PPV	AR%	
Low	Low	Reference	1182	86	1.0					
Low	High	ORe^†^	531	55	1.4	(1.00-2.0)		9%	30%	
High	Low	ORp^†^	584	81	1.9	(1.4-2.6)		12%	48%	
High	High	ORpe^†^	290	57	2.7	(1.9-3.9)		16%	63%	
Total			2587	279						
			Expected ORpe		Departure from		% of ORpe attributable			
			assuming no joint action (E)		expected (DE)		to the joint action of			
							person and environment			
	Additive model of interaction		ORe+ORp-1		ORpe-E		DE/ORpe			
			2.3		0.4		14%			

^†^OR: odds ratio, 95% CI: 95% confidence interval, ORe: lnfant stress exposure, ORp: infant temperament, ORpe: infant temperament and stress exposure, Reference: neither exposure; AR%: attributable risk percent, PPV: positive predictive value.

**Table 5:** Odds ratios for anxiety and depressive symptoms at age 4 years attributable to person-environment interaction between early life stress and impulsive temperament style.

			Anxiety and Depressive Symptoms at 4 years								
Group	Infant impulsivity	Stress exposure		Noncase	Case	OR^†^	95% CI^†^		PPV	AR%	
low/low	low	Low	Reference	1079	94	1.0					
low/high	low	High	ORe^†^	515	62	1.4	(1.00-2.0)		11%	29%	
high/low	high	Low	ORp^†^	687	73	1.2	(0.90-1.7)		10%	19%	
high/high	high	High	ORpe^†^	305	50	1.9	(1.30-20.8)		14%	48%	
	Total			2586	279						
				Expected ORpe		Departure from		% of ORpe attributable			
				assuming no joint action (E)		expected (DE)		to the joint action of			
								person and environment			
		Additive model of interaction		ORe+ORp-1		ORpe-E		DE/ORpe			
				1.7		0.2		11%			

^†^OR: odds ratio, 95% CI: 95% confidence interval, ORe: lnfant stress exposure, ORp: infant temperament, ORpe: infant temperament and stress exposure, Reference: neither exposure; AR%: attributable risk percent, PPV: positive predictive value.
